# Genetic Gains in Yield and Yield Related Traits under Drought Stress and Favorable Environments in a Maize Population Improved Using Marker Assisted Recurrent Selection

**DOI:** 10.3389/fpls.2017.00808

**Published:** 2017-05-17

**Authors:** Folusho Bankole, Abebe Menkir, Gbadebo Olaoye, Jose Crossa, Sarah Hearne, Nnanna Unachukwu, Melaku Gedil

**Affiliations:** ^1^Maize Improvement Programme, International Institute of Tropical AgricultureIbadan, Nigeria; ^2^Plant breeding and Genetics, Department of Agronomy, University of IlorinIlorin, Nigeria; ^3^Biometrics and Statistics Unit, International Maize and Wheat Improvement Center (CIMMYT)Mexico City, Mexico; ^4^Seed maize, International Maize and Wheat Improvement Center (CIMMYT)Mexico City, Mexico; ^5^Bioscience Unit, International Institute of Tropical AgricultureIbadan, Nigeria

**Keywords:** maize, genetic gain, drought tolerance, marker assisted recurrent selection, allele frequency

## Abstract

The objective of marker assisted recurrent selection (MARS) is to increase the frequency of favorable marker alleles in a population before inbred line extraction. This approach was used to improve drought tolerance and grain yield (GY) in a biparental cross of two elite drought tolerant lines. The testcrosses of randomly selected 50 S_1_ lines from each of the three selection cycles (C_0_, C_1_, C_2_) of the MARS population, parental testcrosses and the cross between the two parents (F_1_) were evaluated under drought stress (DS) and well watered (WW) well as under rainfed conditions to determine genetic gains in GY and other agronomic traits. Also, the S_1_ lines derived from each selection types were genotyped with single nucleotide polymorphism (SNP) markers. Testcrosses derived from C_2_ produced significantly higher grain field under DS than those derived from C_0_ with a relative genetic gain of 7% per cycle. Also, the testcrosses of S_1_ lines from C_2_ showed an average genetic gain of 1% per cycle under WW condition and 3% per cycle under rainfed condition. Molecular analysis revealed that the frequency of favorable marker alleles increased from 0.510 at C_0_ to 0.515 at C_2_, while the effective number of alleles (N_e_) per locus decreased from C_0_ (1.93) to C_2_ (1.87). Our results underscore the effectiveness of MARS for improvement of GY under DS condition.

## Introduction

Maize is a staple crop in sub-Saharan (SSA) Africa consumed by over 300 million people. The projected annual maize demand in SSA had been estimated to be 500 million tons by the year 2020, which will surpass the demand for both wheat and rice (IFPRI, 2000). The rapidly growing population in the SSA has therefore necessitated the need to increase maize production in the region therefore; the development of maize varieties with enhanced performance under biotic and abiotic stresses continues to be an important objective (Boomsma and Vyn, 2008). The average yield of maize are significantly lower in Africa compared with the other parts of the world as a result of a host of abiotic and biotic stress factors that affects the crop (FAO, 2010). Under drought stress (DS) and sub-optimal soil nitrogen conditions, the yield of maize is reduced by up to 80% (Bänziger and Lafitte, 1997; Betran et al., 1997; Bänziger et al., 2006). Several studies showed that ear characteristics diminished and the economic yield of maize decreased significantly under drought (Edmeades et al., 1995; Ti-da et al., 2006; Mohammadai et al., 2012). The change in the climate is expected to increase the occasions of drought in Africa (Williams and Funk, 2011), coupled with the expansion of maize production into drought prone regions.

The common conventional breeding approach for drought tolerance involves exploitation of native genetic variation and uses selection to incorporate better characteristics into adapted genotypes (Xoconostle-Cazares et al., 2011). This breeding method has a proven track record of improving tolerance to abiotic stresses (DTMA, 2015). The relative performance of genotypes in DS and favorable environments has been a common criterion to identify desirable genotypes for unpredictable rainfed conditions (Nouri et al., 2011). Direct selection for grain yield (GY) under drought is often more difficult due to low heritability of this trait under stress conditions (Edmeades et al., 1999; Venuprasad et al., 2007; Ziyomo and Bernardo, 2013). In an effort to get the most ideal drought tolerant genotypes for farmers, research is now emphasizing on combining conventional breeding methods with the use of molecular tools (Mhike et al., 2011) to speed up the development and deployment of improved varieties (Beyene et al., 2016).

Marker assisted recurrent selection (MARS) uses markers for identifying several regions on the genome involved in the expression of complex traits and assembling them within a single cross or across related populations (Ribaut et al., 2010). MARS aims at accumulating a large number of quantitative traits loci (QTL) in a population using a subset of markers that are significantly associated with target traits (Bernardo, 2008). Several QTLs associated with GY and other traits under DS and well watered (WW) conditions have been reported (Veldboom and Lee, 1996; Ribaut et al., 1997; Tuberosa et al., 2002; Almeida et al., 2013; Semagn et al., 2013). MARS can then facilitate rapid accumulation of such favorable alleles linked to QTLs in a breeding population (Eathington et al., 2007). When parents are crossed continually based on their genotypes obtained through the use of molecular markers, a genotype that has accumulated favorable alleles at all it loci can be obtained. When parents are crossed based on their molecular marker genotypes, it might be possible to get an ideal genotype after successive generations of crossings (Ragimekula et al., 2013).

The International Institute of Tropical Agriculture (IITA) under the maize improvement programme has improved a biparental maize population through MARS using single nucleotide polymorphism (SNP) markers to enhance GY and other traits under DS condition. The parents used to develop this bi-parental cross were elite drought tolerant maize inbred lines. Understanding genetic gain from MARS and the associated changes in the frequencies of favorable alleles in this population may facilitate the potential use of this approach in developing parental lines of hybrids for drought affected areas. Eathington (2005) and Crosbie et al. (2006) indicated that the genetic gain achieved through MARS in maize was about twice that of phenotypic selection in some reference populations. In upland cotton, Yi et al. (2004) reported significant improvements in resistance to *Helicoverpa armigera* (cotton bollworm) using MARS. Mayor and Bernardo (2009) compared the usefulness of one and two stage phenotypic selection over MARS in testcrosses of double haploid lines and found that multiple cycles of MARS led to the same level of genetic gain as two-stage phenotypic selection, but at a much lower cost. Beyene et al. (2014) compared the effectiveness of MARS with conventional pedigree selection using hybrids derived from 10 bi-parental maize populations and found higher gain for MARS than that for pedigree selection under both DS and optimum growing conditions. In a separate study, comparing MARS and phenotypic selection, hybrids formed from the top C_1_S_2_ lines of each population improved through MARS produced higher mean GYs than hybrids formed from each of the populations improved using phenotypic selection (Beyene et al., 2015).

Unlike the previous studies that used bulk samples of plants representative of the original and advanced selection cycles of populations improved with MARS for crossing with a common tester (Beyene et al., 2014, 2015) evaluating crosses of randomly selected lines from the original and advanced cycles of MARS populations with a common tester provides an opportunity to examine the potential usefulness of improved populations for developing superior drought tolerant maize inbred lines. There is also a need to assess performance of the MARS approach in improving traits in multiple locations under rainfed conditions which are typical of farmers’ growing conditions. This information is useful to determine the potential of improved cycles as sources of productive inbred lines for developing hybrids adapted to rainfed conditions. Furthermore, molecular markers such as SNP can facilitate monitoring shifts in allele frequencies in response to selection (Baskaran et al., 2009). Assessment of the molecular changes that occur in MARS populations may therefore provide information on the genetic components underlying the response to selection. Several studies that examined changes in allele frequencies in maize populations undergoing MARS found increases in the frequency of favorable alleles (Bernardo, 2008; Mayor and Bernardo, 2009; Massman et al., 2013). The objectives of the present study were, therefore, to determine genetic gains in GY (i) under DS and WW conditions, (ii) in multiple locations under rainfed conditions and, (iii) assess the associated shifts in the frequency of marker alleles after two cycles of MARS in a bi-parental population.

## Materials and Methods

### Development of the MARS Population

A MARS population was developed by crossing two elite drought tolerant maize inbreds (DTPL-W-C7-S2-7-1-1-1-1-B-5-B^∗^4 and Babangoyo/MO17LPA/Babangoyo-23-4-3-3-B^∗^6) selected for desirable agronomic traits, resistance to foliar diseases. The resulting F_1_ was selfed to generate F_2_ bulk seeds, which was grown in 50 rows of 5 m length with a spacing of 0.75 m and self-pollinated to generate 300 F_2:3_ lines. A total of 250 F_2:3_ lines from this population were planted each in a row and crossed to an inbred tester of the opposite heterotic group. The testcrosses were evaluated under DS and WW conditions at the International Institute of Tropical Agriculture (IITA) Ikenne substation during the dry season. Irrigation was withdrawn from 6 weeks after planting up to the harvest to elicit DS at flowering and grain filling stages.

### Genomic Estimated Breeding Value and Selection

Marker effects of the genotyped 250 F_2:3_ lines were calculated using the best linear unbiased prediction (BLUP) model (Meuwissen et al., 2001), which permitted predicting genomic estimated breeding value (GEBV) (Henderson, 1975; Gianola and Fernando, 1986). The GEBV was calculated per marker across all the lines derived from the original population (C_0_) using the BLUP values of GY. Each line was scored 0 or 1 based on the presence or absence of parental allele. The BLUP value per marker was multiplied by the marker score of each line and the resultant values per line were the GEBVs. Significant markers on each chromosome were identified by backward elimination. A relaxed significance level (*P* = 0.10), which was found to maximize the response to MARS (Hospital et al., 1997; Johnson, 2001), was used. The selected C_0_ lines were ranked according to their GEBV values and 10% of the F_2:3_ lines with the highest GEBV values were planted ear-to-row and inter-crossed. Bulk pollen collected from ten plants in each line was used for inter-crossing with other lines. Four ears were harvested in each row and shelled to obtain more than 100 seeds per ear. Equal amounts of seed randomly selected from each of the harvested ears were bulked to form the subsequent cycle (C_1_) which was planted in 20 rows. Leaf samples were collected from each of the plants for genotyping at LGC Genomics for SNP genotyping. Genotyping the C_1_ lines were done using SNP markers that were initially used for genotyping the C_0_ population. GEBV were estimated for all C_1_ individual plants and C_2_ was formed by intermating the selected top 10% of the C_1_ individuals. All recombination were conducted at Ibadan in Nigeria.

### Formation of Testcrosses for Phenotypic Evaluation

In 2013, the original (C_0_) and advanced selection cycles (C_1_ and C_2_) of the MARS population were planted at Ibadan each in 60 rows of 5 m length spaced 0.75 m apart. Several plants were self-pollinated and 120 to 150 S_1_ lines were harvested from each selection cycle and retained. Amongst these, 60 S_1_ lines were randomly selected and were planted along with parental lines of the bi-parental cross and an inbred tester (IWD-SYN-STR-C3-5-1-1-B-B) at Ibadan in 2013 to generate testcrosses. The S_1_ lines and the testers were used as female and male parents, respectively. The pollen obtained male parent was bulked and used to pollinate the emerged silks of several plants in each S_1_ lines. The ears from each testcross were harvested and dried to 12.5% moisture content and shelled.

### Genomic Deoxyribonucleic Acid (DNA) Extraction and Isolation

Genomic DNA was isolated from randomly selected leaf samples from each of the 60 S_1_ lines planted to generate testcrosses and the two parents 2 weeks after planting. DNA extraction was done using a cetyltrimethylammonium bromide (CTAB) based DNA extraction protocol optimized in our laboratory by Azmach et al. (2013). Samples of 10 to 20 leaf disks representing each of the genotypes were cut and were placed into 1.2 ml strip tubes. A 2.4 mm steel grinding balls were placed into each tube before adding the leaf samples. The tubes were covered and the leaf samples were grinded for 1 min using a Genogrinder 2000^®^ instrument (BT and C Inc., Basking Ridge, NJ, USA). A 600 μl 2% CTAB extraction buffer was added into each tube and mixed by shaking in the genogrinder at a speed of 1000 strokes for 1 min. The homogenized samples were then incubated for 30 min in a 65°C water bath to free up the nucleic acids from the leaf tissues and cleanse the DNA from other plant materials such as proteins.

Each tube was centrifuged at 3500 rpm for 15 min after adding chloroform isoamyl alcohol (24:1) into each tube and centrifuged at 3500 rpm for 15 min. Subsequently about 200 μl of the supernatant was transferred to new tubes and 400 μl iso-propanol was added. The two solutions were mixed by inverting the tubes gently for 2 min to allow precipitation of the DNA. The DNA precipitated was then pelleted by centrifuging at 3500 rpm for 15 min and the iso-propanol supernatant was discarded. A 500 μl of 70% ethanol was added to the DNA pellet and the tubes were centrifuged at 3500 rpm for 15 min to clean the DNA pellet. The pellets were dried, dissolved in 100 μl ultra-pure water and stored at 4°C. DNA quality and concentration were checked by running 2 μl of the diluted DNA sample on 1% agarose gel. DNA quantification was carried out using a NANODROP^®^ spectrophotometer (Thermo Fisher Scientific Inc., Denver, CO, USA). Genomic DNA samples were lyophilized to dry powder and sent to LGC genomics (UK) for SNP genotyping on Kbiosciences’ KASP assay platform (KBioscence—LGC Genomics^1^). The SNP data obtained from this assay was used to assign genotype score to the population.

### Evaluation of Testcrosses under Drought Stress and Well Watered Conditions

A trial consisting of 50 testcrosses of randomly selected S_1_ lines from each of the three cycles of selection, along with testcrosses of each of the parental lines of the bi-parental cross to the same tester (P_1_ × T, P_2_ × T), a cross of the two parental lines (F_1_) and commercial hybrid checks (Oba super 2, Oba super 7, and Oba super 9) were evaluated under DS and WW conditions at Ikenne during the 2014 and 2015 dry seasons. The testcrosses were arranged in a 26 × 6 alpha-lattice design with two replications. Each testcross was planted in single rows of 5 m with 0.75 m space between the rows while the plants were spaced 0.25 m apart within the rows. In the DS trial, irrigation was withdrawn from 5 weeks after planting through harvest to impose DS, whereas the WW trial received irrigation until physiological maturity. A compound fertilizer was applied at the rates of 60 kg N, 60 kg P, and 60 kg K ha^-1^ at the time of sowing and an additional 60 kg N ha^-1^ 4 weeks later. In each trial, gramoxone and atrazine were applied as pre-emergence herbicides at 5.0 l ha^-1^ and subsequent manual weeding was carried out to keep the experiments weed-free.

### Evaluation of Testcrosses in Multiple Locations under Rain Fed Condition

An experiment consisting of 50 randomly selected testcrosses of S_1_ lines from each of the three cycles of selection, along with testcrosses of each of the parental line of the bi-parental cross to the same tester (P_1_ × T, P_2_ × T), a cross of the two parental lines (F_1_) and commercial hybrid checks (Oba super 2, Oba super 7, and Oba super 9) were evaluated under rainfed condition at Ikenne, Saminaka, and Bagauda during the 2014 and 2015 growing seasons. The testcrosses were also arranged in a 26 × 6 α-lattice design with two replications. Each testcross was planted in single rows of 5 m with 0.75 m space between the rows while the plants were spaced 0.25 m apart within the rows. Basal application of a compound fertilizer was applied at the rates of 60 kg N, 60 kg P, and 60 kg K ha^-1^ at the time of sowing and an additional 60 kg N ha^-1^ 4 weeks later. In each trial, gramoxone and atrazine were applied as pre-emergence herbicides at 5.0 l ha^-1^ and subsequent manual weeding was carried out to keep the experiments weed-free.

### Data Collection

Days to anthesis (DP) and silking (DS) were recorded in each of the plot as the number of days from sowing to when half of the plants were shedding pollen grains and showed emerged silks, respectively. Anthesis-silking interval (ASI) was computed as the interval in days between silking and anthesis. Plant height (PH) and ear height (EH) were measured in centimeters as the distance from the base of the plant to the height of the first tassel branch and the node bearing the upper ear, respectively. Plant aspect (PA) was rated on a scale of 1 to 5, where 1 = excellent overall phenotypic appeal and 5 = poor overall phenotypic appeal. Ear aspect (EA) was scored on a scale of 1 to 5, where 1 = clean, uniform, large, and well-filled ears and 5 = rotten, variable, small, and partially filled ears. Visual leaf death (LD) was scored only under drought at 12 WAP on a scale of 1 to 9, where 1 = less than 10% senesced leaf and 9 = more than 80% senesced leaf area below the ear. The number of ears per plant (EPP) was the proportion of total number of ears divided by the number of harvested plants. All ears harvested from each plot were shelled to determine percentage moisture and GY adjusted to 15% moisture.

### Statistical Analyses

Individual analysis of variance was conducted for each trial using a mixed model in SAS version 9.3 (SAS Institute, 2009). Replication, year, environment and incomplete blocks were considered as random effects while cycle, testcrosses (cycle) and checks were considered as fixed effects. Combined analyses of variance (ANOVA) was performed for traits recorded under both DS and WW conditions. The average gain per cycle was calculated by regressing means of testcrosses averaged over years on the number of cycles of selection. The percentage response per cycle was obtained by dividing the linear regression coefficient with the intercept multiplied by 100 (Menkir and Kling, 1999).

PowerMarker Version 3.25 (Liu and Muse, 2005) and Gen ALEX 6.5 (Smouse and Peakall, 2012) were used for the analysis of molecular data. A total of 180 S_1_ lines derived from the three selection cycles and the parental lines were genotyped. Observed allelic and genotypic frequencies for each SNP locus were used to estimate the frequency of the most common alleles, number of alleles, private alleles, observed heterozygosity, and expected heterozygosity.

### Estimation of Favorable Marker Alleles

Favorable marker alleles are those alleles at the quantitative trait loci (QTL) that made positive contribution to GY during initial evaluation of the bi-parental cross. At initial (cycle 0) the frequencies of the bi-allelic loci wa equal to 0.5 and the alleles were coded “1” and “2” (Liu et al., 2014). The favorable and unfavorable alleles were determined by the positive and negative sign of the estimated marker effect, respectively. The marker covariates were coded as 0, 1, or 2 for estimating the effects of the markers. The score allotted to each of the markers depends on the favorable allele frequencies that vary in each generation. The rationale behind allocation of scores is that selection should be performed for a sufficient number of times to ensure that all favorable alleles are fixed (Liu et al., 2015). For the markers in each cycle, the mean, maximum, and minimum allele frequencies of the favorable alleles were estimated using SAS version 9.3 (SAS Institute, 2009).

## Results

Testcrosses of the S_1_ lines derived from C_0_, C_1_, and C_2_ of the MARS population were evaluated at Ikenne for 2 years under both DS and WW conditions. The average yield of the evaluated testcrosses under DS represented 22% of their average yield under WW condition. In the combined analysis of variance under DS condition, year did not significantly affect all the measured traits except EH and LD. No significant difference was observed among the cycles of selection for all agronomic traits, except for PA (**Table 1**) possibly due to the unexpected severe effect of fall army worms attack. Significant difference was observed among the testcrosses (cycle) for GY only. The interactions of year with cycle and testcrosses (cycle) were not significant for almost all the traits recorded under DS condition. In the combined analysis of variance under WW condition, year had significantly affected five of the nine agronomic traits (**Table 2**). Significant differences were not found among the selection cycles for most agronomic traits. The interaction of years with cycles of selection and testcrosses (cycle) was not significant for all measured traits under WW condition, whereas year had a significant effect on days to silking and PH only.

**Table 1:** Mean square (MS) from the combined analysis of variance for grain yield and other agronomic traits of testcrosses of S_1_ lines derived from different cycles of MARS in a population evaluated under drought stress condition.

Source	df	Grain yield	Anthesis silking interval	Days to silking	Days to anthesis	Plant height	Ear height	Ear aspect	Plantaspect	Leafdeath
Year	1	20130503	14.1	311.4	801.5	82544.8	104764.3^∗^	7.6	9.2	310.5^∗∗^
Rep (year)	2	4716279^∗∗^	4.0	1388.1^∗^	1345.9^∗∗^	18252.8^∗∗∗^	3970.8^∗∗∗^	15.6^∗∗∗^	5.1^∗∗^	2.9
Block (year^∗^rep)	100	615287^∗∗∗^	4.5^∗^	237.2^∗^	165.7^∗^	765.7^∗∗∗^	178.1^∗∗∗^	0.5^∗∗∗^	0.5^∗∗∗^	1.2^∗∗∗^
Cycle	2	630590	5.5	64.4	68.6	670.6	189.2	0.7	1.5^∗∗^	4.4
Testcrosses (cycle)	147	225041^∗^	3.0	137.6	95.7	244.1	68.0	0.2	0.2	0.8
Year^∗^cycle	2	816678^∗∗^	5.7	120.4	40.4	340.4	93.0	0.3	0.0	1.2
Year^∗^testcrosses (cycle)	147	170446	2.8	144.2	98.3	224.8	67.9	0.2	0.2	0.7
Error	198	188420	3.3	173.2	125.7	223.6	75.2	0.2	0.1	0.7

*Significant at ^∗^*P* ≤ 0.05, ^∗∗^*P* ≤ 0.01, and ^∗∗∗^*P* ≤ 0.001 levels, respectively.*

**Table 2:** Mean square (MS) from the combined analysis of variance for grain yield and other agronomic traits of testcrosses of S_1_ lines derived from different cycles of MARS in a population evaluated under well watered condition.

Source	df	Grain yield	Anthesis silking interval	Days to silking	Days to anthesis	Plant height	Ear height	Ear per plant	Ear aspect	Plant aspect
Year	1	32869921	145.1	1153.2^∗^	480.3	90029.3^∗∗∗^	15440.8^∗∗∗^	1.8^∗∗∗^	0.01	3.8^∗∗∗^
Rep (year)	2	2897473	0.74	45.7^∗∗∗^	35.2^∗∗^	801.9^∗∗^	343.3^∗∗^	0.02	3.5^∗∗∗^	0.2
Block (year^∗^rep)	100	863549	0.7	3.0^∗^	3.8^∗∗^	322.3^∗∗∗^	115.3^∗∗^	0.01	0.2^∗^	0.2^∗^
Cycle	2	2167191	0.7	8.5	4.3	694.9^∗∗^	134.7	0.01	0.7^∗∗^	0.1
Testcrosses (cycle)	147	954099	0.7	4.1^∗∗∗^	5.4^∗∗∗^	177.1	78.7	0.01	0.1	0.2
Year^∗^cycle	2	553401	0.5	3.1	3.7	51.4	10.9	0.0	0.1	0.3
Year^∗^testcrosses (cycle)	147	738492	0.6	1.9	2.5	134.2	56.5	0.0	0.1	0.2
Error	198	794249	0.6	2.1	2.6	160.6	66.3	0.01	0.1	0.2

*Significant at ^∗^*P* ≤ 0.05, ^∗∗^*P* ≤ 0.01, and ^∗∗∗^*P* ≤ 0.001 levels, respectively.*

### Genetic Gain in Grain Yield under Drought Stress and Well-Watered Conditions

The overall mean GY of the MARS population under DS was 1032 kg ha^-1^ (**Table 3**). The testcrosses involving S_1_ lines derived from C_2_ produced higher mean GY than those derived from C_0_ with a relative genetic gain of 7% per cycle. Mean GY of the testcrosses at C_2_ was also slightly higher than the parental testcrosses with the same tester and the commercial hybrid checks. The highest mean GY was recorded for the cross between the two parental lines (F_1_). The testcrosses of C_2_ did not differ significantly from other selection cycles for days to flowering, plant and EHs, but differ significantly (ρ > 0.05) for plant and aspect scores. Under WW condition, GY of the testcrosses of S_1_ lines derived from C_2_ was higher than those derived from C_0_, C_1_, the commercial hybrid checks, one of the parental testcrosses and the cross of the two parents (**Table 4**). The average genetic gain in GY for the testcrosses under WW condition was 43 kg per cycle representing a relative gain of 1% per cycle. The gains for other traits were either zero or small.

**Table 3:** Mean grain yield and other agronomic traits of testcrosses of C_0_, C_1_, and C_2_, parents (P_1_, P_2_), first filial generation (F_1_) and commercial checks for the MARS population evaluated under drought stress in 2014 and 2015.

Entries	Grain yield (kg ha^-1^)	Anthesis silking interval (days)	Days to silking (days)	Days to anthesis (days)	Plant height (cm)	Ear height (cm)	Plant aspect (1–5)	Ear aspect (1–5)	Leaf death (1–9)
Cycle 0	972	4	60	56	135	65	3	4	6
Cycle 1	1009	3	60	57	130	62	3	4	6
Cycle 2	1090	3	59	56	135	64	3	3	6
Parent 1 × tester	1079	2	59	57	129	62	3	4	6
Parent 2 × tester	1012	5	61	56	119	59	3	4	5
Parent 1 × 2	1621	3	58	55	133	64	3	3	5
Commercial hybrid checks	881	3	62	59	134	62	3	4	6
Mean	1032	3	60	56	133	64	3	3	6
Average gain per cycle	59	-0.5	-0.5	0.0	0.0	-0.5	0.0^∗^	-0.5	0.0
LSD_(0.05)_	121.1	0.36	2.60	2.21	2.95	1.71	0.07	0.09	0.0
CV	45	56	22	21	11	14	13	13	13

*ns, not significantly different at ρ ≤ 0.05. LSD_(*0.05*)_ = least significant difference at 5% probability level.*

**Table 4:** Average gain, mean grain yield and other agronomic traits of testcrosses of C_0_, C_1_, C_2_, parental testcrosses (P_1_, P_2_), F_1_ and checks for the MARS population evaluated under well watered conditions in 2014 and 2015.

Entries	Grain yield (kg ha^-1^)	Anthesis silking interval	Days to mid silking	Days to mid anthesis	Plant height	Ear height	Ear per plant	Ear Aspect	Plant Aspect
		(days)	(days)	(days)	(cm)	(cm)	(number)	(1–5)	(1–5)
Cycle 0	4612	1	58	57	199	100	0.90	3	2
Cycle 1	4693	1	57	56	201	101	0.90	3	2
Cycle 2	4698	1	58	56	202	100	0.88	3	2
Testcross of Parent 1	2407	0	62	61	199	102	1.03	3	2
Testcross of Parent 2	4801	1	58	57	201	101	0.86	2	2
Parent 1 × Parent 2	3229	1	57	56	198	102	0.85	2	2
Commercial checks	4613	2	59	57	204	100	0.93	3	2
Mean	4644	1	58	56	201	103	0.91	3	2
Average gain per cycle	43	0.0	0.0	-0.5	1.5^∗^	0.0	-0.01	0.0^∗^	0.0
LSD_(0.05)_	180.1	0.15	0.29	0.32	2.50	1.61	0.02	0.07	0.08
Coefficient of variation	19	70	3	3	6	8	12	14	18

*^∗^*P* ≤ 0.05.*

### Genetic Gain in Grain Yield under Rainfed Condition

A separate study was conducted to evaluate these testcrosses at three locations for 2 years under rainfed condition to determine genetic gains in GY and other agronomic traits. The testcrosses involving S_1_ lines derived from C_2_ produced significantly higher GY than those derived from C_0_ with a relative genetic gain of 3% per cycle (**Table 5**). Mean GY of the testcrosses at C_2_ was also higher than the cross of each parent to the tester, the hybrid between the two parents and the average of commercial hybrid checks. The top five testcrosses selected from each selection cycle under DS and their corresponding mean GYs under WW and rainfed conditions are presented in **Table 6**. Testcrosses involving S_1_ lines derived from C_2_ had higher GY than those derived from C_0_ and C_1_ particularly under DS conditions. The best testcrosses from all selection cycles out-yielded the best commercial hybrid check under DS condition by 6 to 44% and the best parental testcross by 22 to 67%. The best testcrosses selected from each selection cycle under well-watered and rainfed conditions were competitive with the commercial hybrid checks in terms of GYs.

**Table 5:** Average gain, mean grain yield, and other agronomic traits of testcrosses of S_1_ lines derived from three cycles of selection through MARS evaluated at three locations in Nigeria in 2014 and 2015.

Entries	Grain yield (kg ha^-1)^	Anthesis silking interval	Days to silking	Days to anthesis	Plant height	Ear height	Ear per plant	Ear aspect	Plant aspect
		(days)	(days)	(days)	(cm)	(cm)	(number)	(1–5)	(1–5)
Cycle 0	5323	1	58	57	188	90	1	3	2
Cycle 1	5503	1	58	57	189	93	1	3	2
Cycle 2	5642	1	58	57	193	93	1	3	2
Testcross of parent 1	2637	0	60	59	197	100	1	3	2
Testcross of parent 2	5535	1	58	57	176	85	1	3	2
Parent 1 × parent 2	3816	0	56	56	190	90	1	2	2
Commercial hybrid check	4944	1	60	58	204	101	1	3	2
Mean	5455	1	58	57	190	92	1	3	2
Average gain per cycle	159^∗∗∗^	0.0	0.2^∗∗^	0.1^∗∗∗^	2.6^∗∗∗^	1.7	-0.004	-0.1^∗∗∗^	-0.02
LSD_(0.05)_	109.74	0.07	0.17	0.16	1.18	0.95	0.02	0.04	0.10
Coefficient of variation	18	63	3	2	5	9	21	13	30

*Significant at ^∗∗^*P* ≤ 0.01 and ^∗∗∗^*P* ≤ 0.001 levels, respectively.*

**Table 6:** Mean grain yield of the five highest yielding testcrosses in each selection cycle and the three hybrid checks under drought stress (DS) and well-watered (WW) conditions.

	Testcross	Drought stress	Well watered	Rainfed
		Grain yield (kg ha^-1^)	Grain yield (kg ha^-1^)	Grain yield (kg ha^-1^)
Cycle 0	24	1739	3779	5059
	14	1435	4820	5415
	21	1402	5871	5683
	25	1378	4725	5423
	39	1367	4651	5158
Cycle 1	80	1757	4526	5487
	79	1597	4444	5528
	86	1509	4590	5323
	51	1452	4905	5738
	52	1445	4425	5156
Cycle 2	112	1876	4172	5119
	126	1789	4925	6033
	136	1732	4682	6066
	103	1638	4108	5439
	119	1622	4405	5391
Parental testcrosses	Parent 1 × Tester	1121	2407	2848
	Parent 2 × Tester	1003	4801	5741
Commercial hybrid check	Oba super 2	274	4029	4064
	Oba super 7	1074	4898	5621
	Oba super 9	1295	4913	5225
	Mean	1032	4644	5455
	LSD (_0.05_)	621	1226	942

### Changes in the Frequencies and Number of Favorable Alleles

Genotyping of randomly selected 180 S_1_ lines derived from the three selection cycles (C_0_, C_1_, and C_2_) and two parental lines of the MARS population using SNP markers showed that the mean frequencies of the favorable marker alleles for GY increased slightly from C_0_ to C_2_ (**Table 7**). The minimum number of favorable marker alleles in C_0_ was higher than those of C_1_ and C_2_ with a difference of 38% at C_1_ and 30% at C_2_. The maximum number of combinations of favorable alleles present in the S_1_ lines derived from C_1_ and C_2_ was higher than those derived from C_0._ The changes in the maximum number of combinations of favorable alleles found in S_1_ lines derived from the original and advanced cycles did not follow a consistent trend. On the other hand, the average number of combination of alleles present in the best 10 lines increased slightly from C_0_ to C_2_ (**Table 8**).

**Table 7:** Mean, minimum, and maximum frequencies of favorable marker alleles associated with grain yield in a MARS population.

Frequency	Cycle 0	Cycle 1	Cycle 2	LSD_(0.05)_
Minimum	0.275	0.144	0.175	0.012
Maximum	0.693	0.839	0.855	
Mean	0.510 ± 0.004	0.512 ± 0.004	0.515 ± 0.004	

**Table 8:** Mean, minimum, and maximum combinations of favorable marker alleles detected in all S_1_ and selected best 10 lines at each selection cycle.

Entry	Minimum favorable alleles	Maximum favorable alleles	Mean favorable alleles
C_0_S_1_ lines	56	129	105 ± 1.5
Best 10 C_0_S_1_ lines	117	129	120 ± 1.2
C_1_S_1_ lines	77	125	107 ± 1.1
Best 10 C_1_S_1_	114	125	118 ± 1.1
C_2_S_1_ lines	73	129	108 ± 1.6
Best 10 C_2_S_1_ lines	119	129	124 ± 1.0
LSD_(0.05)_	4.2		

The changes in the effective number of alleles, level of heterozygosity, and inbreeding coefficient are presented in **Table 9**. The number and the frequency of different alleles were similar across cycles but the number of effective alleles was higher at C_0_ than at C_2_. The level of heterozygosity decreased from 0.591 at C_0_ to 0.500 at C_2_ whereas the average expected heterozygosity (H_e_) slightly increased from 0.458 at C_0_ to 0.478 at C_2_. H_o_ was higher than the H_e_ for all the cycles. As shown in **Table 10**, five genotypes were lost at C_1_ and nine were lost at C_2_.

**Table 9:** Changes in the effective number of alleles and level of heterozygosity across the MARS population.

Population		Number of effective alleles	Observed heterozygosity	Expected heterozygosity	Unexpected unbiased heterozygosity	Fixation index
C_0_	Mean	1.934 ± 0.009	0.478 ± 0.005	0.591 ± 0.008	0.483 ± 0.005	-0.235 ± 0.011
C_1_	Mean	1.878 ± 0.013	0.460 ± 0.005	0.476 ± 0.008	0.464 ± 0.005	-0.032 ± 0.014
C_2_	Mean	1.869 ± 0.012	0.458 ± 0.005	0.500 ± 0.008	0.462 ± 0.005	-0.095 ± 0.012

**Table 10:** Genotypes lost during selection in the marker assisted recurrent selection population.

C_1_ (Markers)	Genotypes	C_2_ (Markers)	Genotypes
PHM4905_6	AA	PHM1766_1	GG
PZA00007_1	CC	PZA00225_8	AA
PZA03603_1	TT	PZA1369_1	TT
PZA03605_1	AA	PZA01715_1	AA
PZB02058_1	TT	PZA01715_2	TT
		PZA2197_1	GG
		PZA02252_1	AA
		PZB00221_3	GG
		PZB02058_1	TT

## Discussion

In the present study, the intensity and timing of DS imposed was severe to elicit differential responses of the testcrosses of S_1_ lines. The observed yield under DS relative to WW condition fell within the range of 20 to 30% which is considered to be severe DS (Banziger et al., 2000). The significant difference in GY among the testcrosses (cycles) under DS condition indicated that MARS was effective in shifting the frequencies of favorable alleles associated with this trait. The observed significant genetic variation among testcrosses may thus provide us with an opportunity to select maize lines with high yield and acceptable agronomic traits. The absence of interaction of year with testcrosses (cycle) for GY and other traits under DS indicated a consistent performance of the testcrosses across years.

The observed higher mean GY of the testcrosses at C_2_ over that at C_0_, parental testcrosses and commercial checks under DS and well-watered condition suggest that MARS was effective for the improvement of complex traits such as tolerance to DS consistent with the findings of Beyene et al. (2015, 2016). Testcrosses of C_2_ did not differ significantly from C_0_ in PH and days to anthesis under DS and WW conditions, indicating that the increase in GY at C_2_ was not associated with an increase in flowering and PH. Previous studies across diverse tropical maize populations (Beyene et al., 2015; Semagn et al., 2015) have also reported a similar trend in which the superiority of MARS was demonstrated without significantly affecting PH and maturity of most populations. The higher GY recorded for the parental testcrosses (F_1_) under DS condition for 2 years may be related to the superiority of the highly homogeneous F_1_ relative to the heterogeneous testcrosses involving S_1_ lines derived from C_0_, C_1_, and C_2_.

The changes in the frequencies and combinations of favorable alleles with selection though not significant, showed that MARS experienced a slight but consistent increase in accumulating favorable alleles for GY. The gradual increases in mean frequencies and number of favorable alleles for the MARS population are in line with the findings of Mayor and Bernardo (2009) who reported that MARS led to desirable changes in the frequencies of favorable marker alleles. Edwards and Johnson (1994) had earlier reported increase in the frequencies of favorable alleles from 0.5 to 0.8 in a sweet corn F_2_ population. Eathington et al. (2007) also reported changes in the frequencies of favorable alleles from 0.56 to 0.96 in 20 different QTL regions, which significantly increased the possibility of obtaining a desirable genotype.

The maximum number of effective alleles (N_e_) is 2 with bi-allelic SNP markers. The fact that the average N_e_ decreased from 1.934 in the starting population to 1.869 in C_2_ suggests that this population is gradually moving toward allele fixation (Vogel, 2010). The reduction in the level of heterozygosity and an increase in the values of the fixation index from C_0_ to C_2_ imply loss of heterozygosity possibly because of positive assortative mating and selection of homozygotes (Smouse and Peakall, 2012). The observed decrease in the number of heterozygotes and the increase in the level of inbreeding resulting from response to selection were in line with the findings of Massman et al. (2013). The loss of genotypes suggests that there were genetic changes in the population due to inadvertent selection against those homozygote alleles. Nonetheless, the loss of these genotypes had no adverse effect on improvements in GY and other traits.

In summary, this study examined the genetic gain in a bi-parental cross of elite drought tolerant lines with good agronomic traits, resistance to maize streak virus disease and other foliar diseases that had undergone two cycles of MARS. The population did respond to MARS for improvement in GY and other agronomic traits. These findings highlight the effectiveness of MARS in improving bi-parental populations as potential success of improved drought tolerant inbred lines with superior agronomic traits under drought condition.

## Author Contributions

FB: Acquisition of data, analysis and interpretation of both phenotypic and molecular aspects of the work, and drafted the work. AM: Designed the work, revised it critically for intellectual content and approval of final version to be published. MG: Designed and supervised the molecular aspect of the work, revised the aspect of the work critically for intellectual content and also approved the final version to be published. GO: Supervision of the field and quantitative genetics aspect of the work, contributed to revising it critically for intellectual content and approval of final version to be published. JC: Analysis of the molecular data during the development of the population, designed the form of selection to be used, revised and approved the final version to be published. SH: Substantial contribution to the design, analysis and interpretation of molecular data while developing the population. NU: Acquisition, analysis, and interpretation of molecular data.

## Conflict of Interest Statement

The authors declare that the research was conducted in the absence of any commercial or financial relationships that could be construed as a potential conflict of interest.

## Footnotes

1 http://www.kbioscience.co.uk/
